# Correction: Khavinson et al. Neuroprotective Effects of Tripeptides—Epigenetic Regulators in Mouse Model of Alzheimer’s Disease. *Pharmaceuticals* 2021, *14*, 515

**DOI:** 10.3390/ph18010111

**Published:** 2025-01-16

**Authors:** Vladimir Khavinson, Anastasiia Ilina, Nina Kraskovskaya, Natalia Linkova, Nina Kolchina, Ekaterina Mironova, Alexander Erofeev, Michael Petukhov

**Affiliations:** 1 Saint Petersburg Institute of Bioregulation and Gerontology, 197110 Saint Petersburg, Russia; vladimir@khavinson.ru (V.K.); miayy@yandex.ru (N.L.); katrine1994@mail.ru (E.M.); 2 Pavlov Institute of Physiology of Russian Academy of Sciences, 199034 Saint Petersburg, Russia; 3 Institute of Biomedical Systems and Biotechnology, Peter the Great St. Petersburg State Polytechnic University, 195251 Saint Petersburg, Russia; ninakraskovskaya@gmail.com (N.K.); alexandr.erofeew@gmail.com (A.E.); 4 Petersburg Nuclear Physics Institute Named after B.P. Konstantinov, NRC “Kurchatov Institute”, 188300 Gatchina, Russia; ininakolchina@mail.ru (N.K.); michael.petukhov@yandex.ru (M.P.); 5 Russian Scientific Center of Radiology and Surgical Technologies Named after A.M. Granov, 197758 Saint Petersburg, Russia

In the original publication [1], there was a mistake in Figures 5 and 8 as published. The mistake is that Figures 5 and 8 are the same, described differently. The corrected Figure 5 and Figure 8 appear below. The authors state that the scientific conclusions are unaffected. This correction was approved by the Academic Editor. The original publication has also been updated.

## Figures and Tables

**Figure 5:**
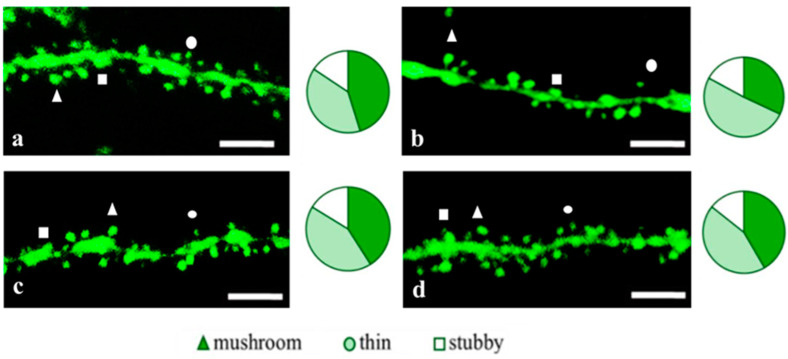
Confocal images of the CA1 secondary dendrites in 5 month-old male M-line (**a**) and 5xFAD-M mice, injected with a physiological solution (**b**), EDR peptide (**c**), KED peptide (**d**). Confocal microscopy, ×100. Scale bar, 10 μm.

**Figure 8:**
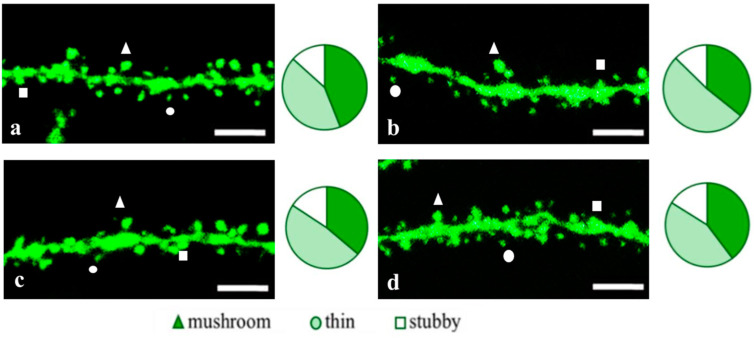
Confocal images of the CA1 secondary dendrites in 5 month-old female M (**a**) end 5xFAD-M mice injected with a physiological solution (**b**), EDR peptide (**c**), KED peptide (**d**). Confocal microscopy, ×100. Scale bar, 10 μm.
